# Healthy Immunity on Preventive Medicine for Combating COVID-19

**DOI:** 10.3390/nu14051004

**Published:** 2022-02-27

**Authors:** Pulak R. Manna, Zackery C. Gray, P. Hemachandra Reddy

**Affiliations:** 1 Department of Internal Medicine, Texas Tech University Health Sciences Center, Lubbock, TX 79430, USA; zackery.gray@ttu.edu (Z.C.G.); hemachandra.reddy@ttuhsc.edu (P.H.R.); 2 Neuroscience & Pharmacology, Texas Tech University Health Sciences Center, Lubbock, TX 79430, USA; 3 Neurology, Departments of School of Medicine, Texas Tech University Health Sciences Center, Lubbock, TX 79430, USA; 4 Public Health Department of Graduate School of Biomedical Sciences, Texas Tech University Health Sciences Center, Lubbock, TX 79430, USA; 5 Department of Speech, Language and Hearing Sciences, School Health Professions, Texas Tech University Health Sciences Center, Lubbock, TX 79430, USA

**Keywords:** nutrition, vitamins, immune health, COVID-19, aging and underlying medical conditions, therapeutic strategies

## Abstract

Immunomodulation is influenced by the consumption of nutrients, and healthy immunity is pivotal to defending an individual from a variety of pathogens. The immune system is a network of intricately regulated biological processes that is comprised of many organs, cellular structures, and signaling molecules. A balanced diet, rich in vitamins, minerals, and antioxidants, is key to a strengthened immune system and, thus, crucial to proper functioning of various physiological activities. Conversely, deficiencies of these micronutrients, involving impaired immunity, are linked to numerous health complications, along with a host of pathologies. Coronavirus disease 2019 (COVID-19) is a dangerous infectious disease caused by a β-form of the severe acute respiratory syndrome coronavirus 2 (SARS-CoV-2) and its genomic variants, which enter host cells upon binding to the angiotensin converting enzyme 2 receptors, and is associated with substantial morbidities and mortalities globally. Patients afflicted with COVID-19 display asymptomatic to severe symptoms, occurrences of which are multifactorial and include diverse immune responses, sex and gender differences, aging, and underlying medical conditions. Geriatric populations, especially men in comparison to women, regardless of their states, are most vulnerable to severe COVID-19-associated infections and complications, with fatal outcomes. Advances in genomic and proteomic technologies help one understand molecular events, including host–pathogen interactions and pathogenesis of COVID-19 and, subsequently, have developed a variety of preventive measures urgently, ranging from mask wearing to vaccination to medication. Despite these approaches, no unique strategy is available today that can effectively prevent and/or treat this hostile disease. As a consequence, the maintenance of a boosted immune system could be considered a high priority of preventive medicine for combating COVID-19. Herein, we discuss the current level of understanding underlining the contribution of healthy immunity and its relevance to COVID-19 molecular pathogenesis, and potential therapeutic strategies, in the management of this devastating disease.

## 1. Introduction

The immune system has long been known as the primary preventive measure against invading pathogens. Maintenance of a boosted immune system is dependent upon proper nutrients, which, ultimately, prevent an organism from infections evolved from a variety of environmental toxins, bacteria, and viruses. Epidemiological evidence indicates a strong correlation between the intake of vitamins/micronutrients and a reduction/protection of pathogenic threats, and, thus, the appropriate functioning of a variety of physiological functions [1,2]. Therefore, eating a healthy and balanced diet is known to play an important role in enhancing the immune system and has numerous health benefits, as well as protective effects against the development of diseases [2,3,4]. It is unquestionable that proper nutrition has a positive impact on COVID-19-related infections, involving shorter durations, severity, and outcomes, and it is inversely correlated with the disease pathogenesis. Conversely, nutritional deficiencies and unhealthy diets, involving impaired immunity, are associated with a variety of complications and disorders and are more susceptible to severe COVID-19-associated issues [5,6,7]. As such, a healthy immune system can serve as a preventive medicine against COVID-19-driven complications and mortalities.

COVID-19, one of the most contagious diseases, is caused by severe acute respiratory syndrome coronavirus 2 (SARS-CoV-2), which was first identified in December 2019 at Wuhan, China [8,9]. Since then, this new virus has created a severe health crisis globally, with COVID-19-associated infections and mortalities over 396 and 5.6 million, respectively, in 225 countries and territories, as of 31 January 2022, with the United States leading in both rates (Worldometers.info). Of note, COVID-19 displays similar clinical features to those of SARS (Severe Acute Respiratory Syndrome) and MERS (Middle East Respiratory Syndrome) that have been previously reported [10]. Airborne transmission is the primary mode of infection for the spread of the COVID-19 virus that enters host cells upon binding to the angiotensin converting enzyme 2 (ACE2) receptors [11,12]. The pathophysiological manifestations of this disease include fever, headache, respiratory distress, hypoxia, lung injury, inflammation, and cardiovascular diseases (CVDs). The severity of COVID-19 infections, by affecting the immune system, damages multiple organ systems, leading to fatal outcomes [13,14]. 

Accumulating evidence indicates that aging populations, especially men in comparison to women, are most susceptible to severe complications, including mortalities, from COVID-19 [15,16]. Since no therapies are effective to prevent and/or cure this deadly disease today, healthy diet and/or lifestyle, involving modulation of the immune system, is pivotal to combating COVID-19. Even so, the COVID-19 pandemic, impacting infections and mortalities worldwide, has prompted the development and urgent approval of a number of measures, including vaccinations and medications [17,18]. Currently, a few anti-viral drugs and antibody cocktails have received emergency use authorization (EUA) to respond to and/or treat certain COVID-19 patients to reduce hospitalizations, as well as mortalities [19,20,21,22,23,24,25]. Therapeutic strategies, targeting the prevention and treatment of COVID-19, require an improved understanding of the host–pathogen interacting mechanism(s), symptomatology, and disease pathogenesis. 

## 2. Functional Importance of the Immune System and Its Connection to COVID-19

**Figure 1:**
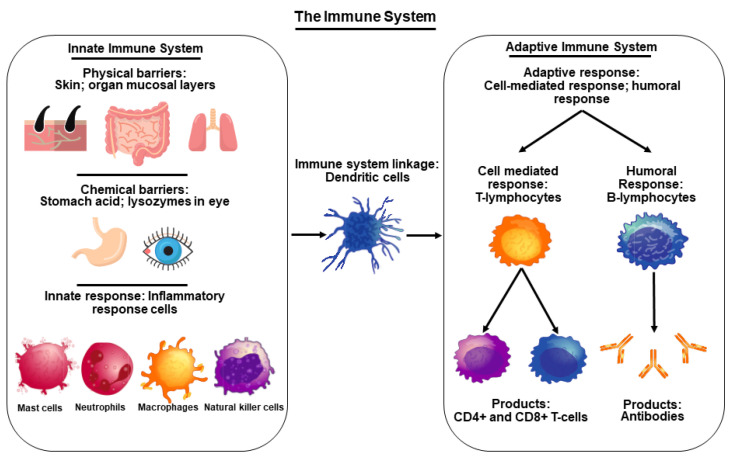
An overview of the immune system illustrating innate and adaptive immune system components. The innate immune system is composed mainly of physical and chemical barriers, and an initial inflammatory response made up of mast cells, neutrophils, macrophages, and natural killer cells. The adaptive immune response works through B- and T-lymphocytes creating a specialized and learned immune response to specific pathogens through antibodies and CD4+ and CD8+ cells. The adaptive and innate components are connected through dendritic cells that allow for the activation of the adaptive response once the innate system has recognized a threat to that organism.

The immune system is a network of intricately regulated biological processes that acts as a barrier between pathogens and the internal milieu. Immune cells originate from bone marrow precursors and develop into mature cells, which play discrete roles for diverse biological activities. Malfunction of the immune system generates many complications, including autoimmune diseases and recurring as well as life-threatening infections. Overall, the immune system detects and responds to a wide variety of pathogens, and it is divided into two parts, i.e., the innate immune system and the adaptive immune system (Figure 1). 

The innate immune system relies upon the physical features and gene expression of an organism and is present at birth. This immune response provides the initial defense against invading pathogens [26]. The innate system involves skin, epithelial tissue linings, respiratory tract, and genitourinary tract, as well as the mucus layers that coat these tissues, and includes neutrophils, monocytes, macrophages, cytokines, and specific proteins [27,28]. Importantly, skin provides a solid and sturdy barrier for the internal milieu against environmental toxins, ultraviolet radiation, bacteria, and germs. Skin is composed of three layers, i.e., outermost epidermis, middle dermis, and innermost hypodermis, essentially a subcutaneous fat layer [3,29,30]. Skin possesses various biomolecules that neutralize pathogenic organisms, including antimicrobial peptides that break down pathogenic membranes [27]. 

The epithelial cells have direct contact with outside molecules such as the inner linings of airways, lungs, and digestive tracts, which are tightly packed and function under a consistently replenished mucus layer to remove foreign matter through literal movements of cilia and other mucus migrating systems [26]. Additionally, neutrophils are the body’s first cellular line of defense for external pathogens that are ingested through phagocytosis and subsequently metabolized [31]. Macrophages, being part of innate response, are capable of engulfing and consuming foreign substances through toll-like receptor mediated mechanisms (Figure 1). Cytokines provide a large influx of blood and immune cells to the sites of infection to combat pathogens [32]. In addition, several enzymes, including lactoferrin and lysozyme, are involved, under innate immune responses, in protecting the body from a variety of invaders.

**Figure 2:**
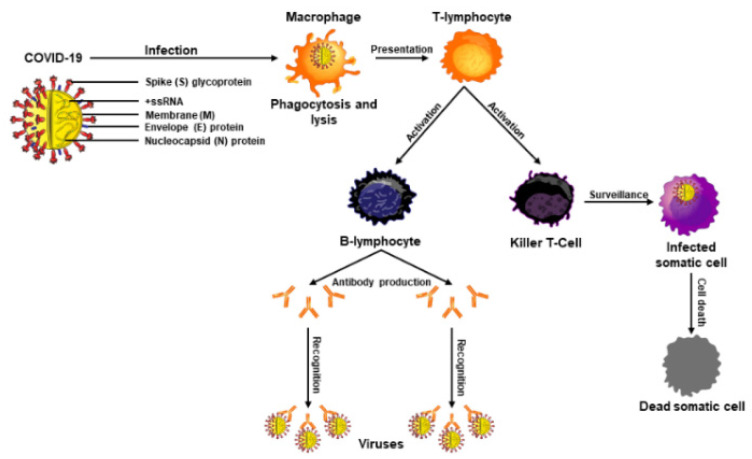
Schematic representation of a COVID-19 virus and its different components, i.e., spike (S), membrane (M), nucleocapsid (N), envelope (E), and RNA structure (+ssRNA). Shown is a proposed mechanism of COVID-19 infection and resultant immune response, including parts from the innate and adaptive immune system. COVID-19 virus enters the body, then macrophages from the innate response are able to ingest some viruses and destroy them from phagocytosis. This releases viral components and antigens, which, in turn, activate the adaptive immune response through T- and B-lymphocytes creating specialized killer T-cells and specific antibodies to combat COVID-19 infections and kill the cells already infected with the virus.

The adaptive or acquired immune system, in coordination with the innate system, is generally evolved to protect an organism exposed to microbes or toxins. This immune process produces antibodies against a specific pathogen or antigen and prevents the body from that specific invader [33]. The adaptive immune response is reserved for complex vertebrates and has shown an evolutionary trend stemming off the innate response. Of note, T- and B-lymphocytes mainly comprise the adaptive immune response, and these immune cells are matured in the thymus and made in the bone marrow, respectively [33]. Mature T-cells influence immunity through cytokine production, antigen destruction, and maintenance of other immune cells [34]. Furthermore, macrophages play an important role in recognizing pathogens, attaching to them, and carrying them to T- and B-lymphocytes for destruction. B-lymphocytes are produced rapidly and serve as memory cells to specific antigens, and once a pathogen is recognized, they work to prevent future infections (Figure 2). This acquired memory is unique to each individual and provides a tailored immune response against the pathogens. B-cells are also responsible for the production of natural antibodies that are explicit to each antigen and attach themselves when that antigen is found within the body [34,35,36].

A heathy immune system is not only the first line of protection but often the best line of defense against pathogens such as viruses, bacteria, and parasites. While balanced food rich in vitamins and minerals helps boost immunity in preventing invaders, nutritional deficiencies result in impaired immunity. In accordance with this, COVID-19 infections and outcomes have been shown to be disastrous among people with weakened immune health, connecting age and age-associated diseases or underlying medical conditions, in contrast to mild to moderate effects with healthy immunity [37,38]. Therefore, the immune system and its functional efficacies have been under intense investigation, which has led to the development of multiple strategies, including nutritional benefits, vaccines, and preventive drugs that ward off pathogens [39]. Nonetheless, vitamins, nutrients, and antioxidants are key factors in strengthening the immune system that positively impact various complications and diseases/infections. Therefore, maintenance of healthy immunity, with various micronutrients, results in long-term health benefits in multiple respects. Known infectious diseases, such as COVID-19, have shown strong implications towards high mortality rates among the elderly and those with age-related diseases, as well as immunocompromised conditions, in which the immune system fails in fighting off different pathogens [37,40]. 

Immunocompromised events include chronic lung and kidney diseases, obesity, dementia and/or neurological disorders, organ transplants, chemotherapy, hematological malignancies, and autoimmune and inherited diseases, all of which enhance COVID-19 susceptibility [7,41,42]. Destruction of T-cells is a common occurrence with immunodeficiency virus, hepatitis B, and acquired immunodeficiency syndrome and/or sexually transmitted diseases. All of these immunocompromised conditions are liable to be influenced not only by COVID-19 infections but also by other pathogens and can lead to fatal consequences. A variety of approaches have targeted immune boosting with vitamins, especially D, C, and E, for patients who are suffering from acute respiratory distress syndromes and other symptoms in conjunction with COVID-19 [18,43]. Accordingly, the immune system is a major focus of modern medicine, which functions as the principle means of prevention for various diseases. A boosted immune response helps keep foreign bodies away by developing physical barriers such as skin and stomach acid, through innate immunity employing phagocytic and killer cells (Figure 2) and through adaptive immunity involving B- and T-lymphocytes. 

## 3. Vitamin and Immune Health Dynamics in Healthy Physiology

**Figure 3:**
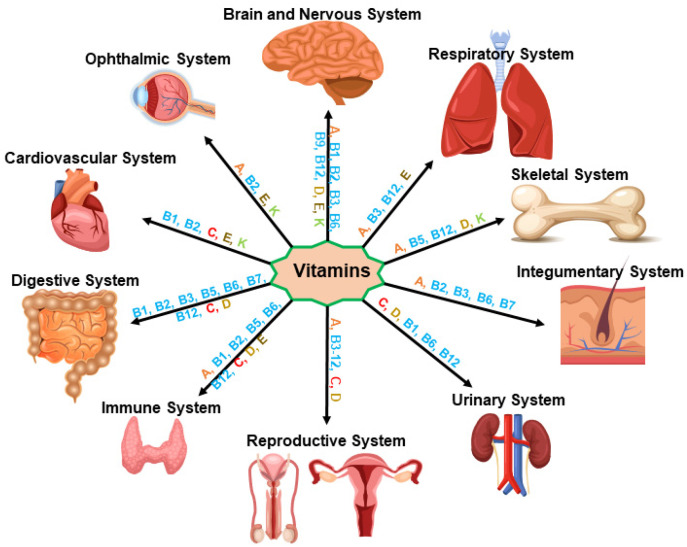
A diagram illustrating the roles of various vitamins on different organ systems. All of these vitamins play important roles in the immune system, in addition to growth and development of various organs and, thus, physiological activities. Organ systems illustrated are the brain and nervous system, respiratory system, skeletal system, integumentary system, urinary system, reproductive system, immune system, digestive system, cardiovascular system, and ophthalmic system. Involvement of different vitamins to a particular organ is shown by a line pointing an arrow to the specific organ system.

Vitamins are essentially required for growth and development, and they play integral roles in the appropriate functioning of the immune system as well as in overall healthy physiology. Vitamins are categorized in two groups: water-soluble (C and eight B vitamins, i.e., B1, B2, B3, B5, B6, B7, B9, and B12) and fat-soluble (A, D, E, and K). Whereas water soluble vitamins are not stored in the body, the latter categories are stored in fat tissues, liver, and muscles. All of these organic compounds are generally obtained from various food sources that people consume regularly, including vegetables, legumes, asparagus, cereals, grains, fruits, berries, nuts, meats, poultry, dairy products, and potatoes, and are essential for healthy immunity in favor of invading pathogens (Figure 3). On the other hand, deficiencies of these micronutrients, involving impaired immunity, result in a variety of health complications and diseases. 

### 3.1. Vitamin A (Retinol)

Vitamin A and its derivatives (retinoids), possessing antioxidant properties, play pivotal roles in the maintenance of both innate and adaptive immune responses and many important biological activities, ranging from vision to reproduction to homeostasis [44,45]. The recommended daily amount (RDA) of vitamin A is 700 µg and 900 µg for adult women and men, respectively. The protective effects of retinoids on a number of viruses, including hepatitis B, influenza, and cytomegalovirus, have been demonstrated [41,46]. Vitamin A deficiency has been shown to be correlated with COVID-19 incidence and mortality [18]. It has been reported that retinoic acid agonist acts on SARS and MERS through the interruption of lipogenic pathways [18,47], which could be used in combination with other antiviral drugs in the management of COVID-19 or other viral infections.

### 3.2. Vitamin B

Vitamin B is essential for appropriate functioning of the immune system [48,49]. Eight vitamins in the B complex are the following: vitamin B1 (Thiamin), vitamin B3 (Niacin), vitamin B5 (Pantothenic acid), vitamin B6 (Pyridoxine), vitamin B7 (Biotin), vitamin B9 (Folic acid, Folate), and vitamin B12 (Cyanocobalamin), which are obtained from various food sources. The RDA of these B vitamins for adult women and men is 1.1 mg and 1.2 mg (B1 and B2), 14 mg and 16 mg (B3), 5 mg (B5), 1.7 mg to 1.3 mg (B6), 20–30 mg (B7), 400 µg (B9), and 2.4 µg (B12), respectively. All of these B vitamins possess antioxidant properties and are required for growth and development, in addition to the maintenance of heathy immunity in the prevention of various pathogens (Figure 3). Notably, vitamin B12, in combination with vitamin B6 and B9, has been targeted as a potential therapy for the management of COVID-19 [50,51].

### 3.3. Vitamin C (Ascorbic Acid or Ascorbate)

Vitamin C has long been known to boost the immune system. It protects against various pathogens and respiratory tract infections, assists in their healing, and decreases and shortens common cold symptoms [52]. The RDA of vitamin C is 75 mg and 90 mg for adult women and men, respectively. Vitamin C reduces not only infections with sepsis and acute respiratory distress syndrome (ARDS) but also various symptoms associated with COVID-19 [53]. Noteworthily, vitamin C displays antioxidant, anti-inflammatory, and immuno-modulatory effects, thus preventing many infections caused by bacteria, viruses, and environmental toxins, and could also be beneficial in the prevention of COVID-19. 

### 3.4. Vitamin D (Ergocalciferol)

Vitamin D is a group of fat-soluble secosteroids, which possess anti-inflammatory, antioxidant, and neuroprotective properties and primarily support immune health and maintain healthy bones [54,55,56]. Vitamin D regulates a number of genetic pathways and influences several health conditions such as cancer, diabetes, respiratory tract infections, and autoimmune diseases. The RDA of vitamin D is 15 µg for both adult women and men. Vitamin D influences both innate and adaptive immune responses; therefore, eating a diet rich in vitamin D might protect people from COVID-19 infections [41,57]. A multicenter study has reported that serum vitamin D levels are markedly decreased in COVID-19 patients when compared with non-infected COVID-19 individuals [58]. Vitamin D supplementation has been shown to reduce acute respiratory infections and enhance the immune system and blood oxygen and hemoglobin levels, thereby lowering the risk of infection, severity, and mortality caused by COVID-19 [55,59]. 

### 3.5. Vitamin E (Tocopherol)

Vitamin E includes four tocopherols and four tocotrienols, which support immune function and play crucial roles in a wide variety of physiological processes, ranging from vision to skin health [60]. Vitamin E possesses antioxidant and anti-inflammatory properties, controls regulation of enzymes involved in a number of signal transduction pathways, and increases lymphocyte and IL-2 proliferation [61,62,63]. Importantly, vitamin E neutralizes reactive oxygen species (ROS) and, by doing so, it plays an important role in protection from heart diseases and certain cancers, in addition to blocking lung neutrophil inflammation (Figure 3). The RDA of vitamin E is 15 mg for both adult men and women. Considering the various beneficial effects of vitamin E, elderly individuals are encouraged to intake this nutrient for the prevention of COVID-19 infection. 

### 3.6. Vitamin K (Phylloquinone)

Vitamin K generates proteins involved in blood coagulation, blood calcium regulation (prothrombin), bone mineralization, and bone health (osteocalcin). The RDA for vitamin K varies upon age and gender; however, the values are generally 90 µg and 120 µg for adult women and men, respectively. Vitamin K decreases production of proinflammatory cytokines and reduces atherosclerotic calcification and/or lesions by 50%, which may be beneficial in decreasing the severity of symptoms and fatal outcomes associated with COVID-19 [64,65,66]. Additionally, vitamin K insufficiency has been reported in COVID-19 patients [67]; thus, vitamin K may be influential in decreasing the severity of symptoms and fatal outcomes of this hostile disease.

## 4. COVID-19: Epidemiology, Risk Factors, and Molecular Pathogenesis

COVID-19, an acute respiratory disease, is caused by a β virus, with clinical features essentially similar to SARS (Severe Acute Respiratory Syndrome) and MERS (Middle East Respiratory Syndrome), with highly conserved genomic configurations. Epidemiological evidence indicates that the majority of COVID-19 cases involve asymptomatic to moderate symptoms, whereas a small group of patients develop critical signs, including pneumonia, ARDS, sepsis, and multiple organ failure [68,69,70]. The WHO has declared certain strains of COVID-19 as Variants of Concern (VOCs), variants that have increased COVID-19 transmissibility, severity, epidemiology, and clinical disease presentation, or have decreased the effectiveness of current treatments options [71]. COVID-19-associated infections and mortalities are considerably higher in elderly populations, especially with men in comparison to women, due to a variety of factors. It is noteworthy that a large number of COVID-19 patients recover from viral infections due to their healthy immunity. As such, children and adolescents, possessing healthy immunity, generally exhibit mild symptoms, including fever, headache, fatigue, and nasal congestion [12,72]. Conversely, a subset of patients, with impaired immunity and/or underlying medical conditions, display severe clinical manifestations, requiring hospitalizations and life-supporting treatments, with fatal outcomes [73]. COVID-19 infections, aided by different genomic variants, are still burdensome to nearly every country, leading to the implementation of a wide variety of measures, including social distancing, mask wearing, vaccinations, and many EUA drugs and/or antibodies to control this hostile disease.

COVID-19 etiology includes human-to-human transmission, including direct contact and airborne transmission, along with a variety of risk factors that involve activities, procedures, products, and events. The contribution of these risk factors is dependent on age, gender, demographics, and immune health, in which aging populations, compared to younger individuals, are more susceptible to COVID-19 infections (Table 1).

The COVID-19 virus encompasses four structural proteins: spike (S), membrane (M), envelop (E), and nucleocapsid (N), and enters the host cells through endocytosis involving three steps, binding, cleavage, and fusion [9]. This virus binds to the ACE2 receptors present in many tissues, with a higher prevalence within the lungs, heart, and kidneys. Notably, COVID-19 genomic variants, i.e., α, β, γ, δ, and Omicron (most contagious), have shown significant effects in different parts of the world and are responsible for the majority of COVID-19-associated infections and complications. Mechanistically, the spike protein is composed of two functional subunits, S1 and S2; the former binds to ACE2, and S2 is responsible for viral fusion [12,74]. The binding of the S2 subunit allows for insertion of the RNA genome into the host cells, which then undergoes proteolytic cleavages by host proteases (e.g., furin and trypsin) and translation to form polyproteins that are then assembled to make replication-transcription complexes. Once the complex is formed (a copy of the RNA genome), structural proteins are synthesized in the cytoplasm and assembled with help from the endoplasmic reticulum and Golgi apparatus [12,74,75]. The viral particles are then released from the cell by exocytosis and have the ability to infect other cells and continue the replication process. 

## 5. Sex and Gender Differences and Their Relevance to COVID-19

**Figure 4:**
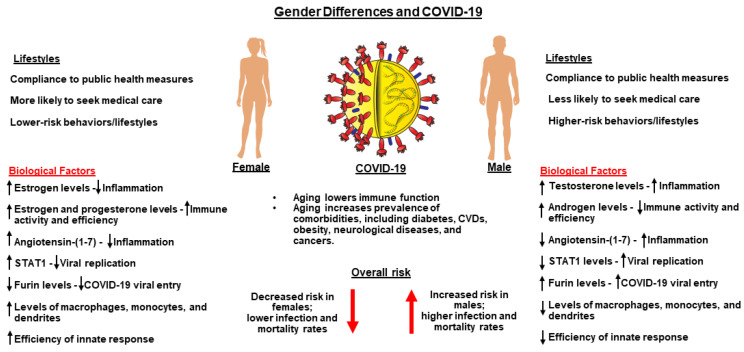
Sex and gender differences and their connection to COVID-19. Several biological factors demonstrate a predisposition of males for higher levels of testosterone and androgen, less efficient innate immune responses, and less ability to repair damages, thus allowing for higher severe infection and mortality rates than their female counterparts. Females demonstrate certain factors such as the presence of estrogens and progesterone, more effective innate immune responses, and other conditions that limit COVID-19′s entry and subsequent infection. Independent of gender, age plays an important role in disease severity. Hormonal balances, lowered immune function, and increased comorbidity prevalence associated with age present opportunities for higher mortality rates and increased disease severity.

An overwhelming amount of evidence indicates that COVID-19 has considerable sex and gender disparities in conjunction with infections, hospitalizations, and mortalities [76,77]. Initially, COVID-19 infections were thought to have similar susceptibility to both men and women; however, clinical findings, connecting severity and fatality, are strikingly higher in males than females [78,79], which could be due to distinctions of male vs. female adaptive and innate immune responses, hormonal differences, social/behavioral habits, and comorbidities (Figure 4). Another study in Europe, on the impact of sex and gender differences, has demonstrated a significantly higher number of deaths involving COVID-19 in males than females [80]. A meta-analysis of 3,111,714 global COVID-19 cases showed a ratio of 3:1 males to females who required intensive treatment and therefore had higher probabilities of death [81]. While there is no precise relationship between males and females with COVID-19, the numbers trend toward males being more likely to develop severe complications and hospitalizations, with fatal outcomes [77,82,83]. In support of this, more women than men generally received COVID-19 vaccines, and women are more likely to involve prevention measures and be compliant regarding mask wearing and social distancing [84].

The immune system differs in function between males and females, such that the latter group generally possesses a stronger adaptive immune response, compared to those of males, possibly due to sex- and gender-based immunological differences, including the enhanced production of antibodies [85]. Moreover, the gender disparities regarding the innate immune response could be due to higher amounts of cytokine IFN-I, including sex hormones, in women compared to men mainly due to the suppression of IFN-I by testosterone [86]. Differences in sex hormones show a general influence upon the immune system (Figure 4). Estrogen possesses anti-inflammatory processes that speed tissue recovery following infection or injury and can provide quicker paths for the female body to terminate the innate inflammatory response [87]. This could further reduce the cytokine storm and better protect women against tissue and organ damages, explaining gender differences associated with severe COVID-19 infections. The signal transducer and activator of transcription 1 (STAT1) are responsible for upregulating certain antiviral gene expressions and are induced by IFN α and β, which have shown to evade upregulation in the presence of a SARS infection [76]. Estrogen is a known up-regulator of STAT1 signaling and provides females with an inherently more robust antiviral immune defense [76]. Conversely, furin is expressed on the cell membrane that assists COVID-19 viral entry and has presented higher expression in males (Figure 4). Furthermore, there are differences between men and women regarding the renin-angiotensin aldosterone system, and women overall show higher rates angiotensin II (Ang II) metabolism, leading to higher rates of Ang (1–7) within the bloodstream plasma [88]. Vasoconstriction occurs in the presence of Ang II; thus, females’ general condition of less Ang II is thought to produce fewer instances of inflammation and a better defense against inflammation such as ensues with a severe COVID-19 infection [76].

Behavioral aspects could provide other indications as to why the disparity of COVID-19 infections and mortalities are higher in males. In accordance with this, smoking and COPD (chronic obstructive pulmonary disease) are associated with increased ACE2 receptor expression in the lungs, a high-risk factor for COVID-19 infection. Males are often accompanied by higher rates of certain COVID-19 comorbidities such as CVDs, hypertension, and diabetes, which are known to increase severe COVID-19-associated complications and mortalities [89]. 

There is increasing evidence that age is likely the most significant risk factor regarding disease severity with COVID-19 [90]. Studies have reported that COVID-19-driven mortality rates are higher among males in all age groups 20 years or older, with a mortality rate two times higher in men than women [81,91]. A retrospective study, based on age and gender, has demonstrated that males and females at 0–17 years of age do not display a significant difference in terms of the number of COVID-19 infections, but at higher ages, males are 64% more vulnerable to severe illnesses and death. The potential mechanisms accounting for the differences in COVID-19-associated infections, severity, and mortalities in males and females could be multifactorial, including aging, social and behavioral habits, physiological differences, and immune-endocrine processes (Figure 4). 

## 6. Aging, Underlying Medical Conditions, and Their Correlation to COVID-19

Aging is an inevitable heterogeneous phenomenon, and it affects the structural and functional properties of a multitude of organs [92,93,94]. The occurrence of hormone deficiencies is connected with human senescence, resulting in a variety of complications and diseases, including diminished eyesight, impaired memory and cognitive function, increased risk of CVDs, and skin disorders [42,95,96,97]. The function of the immune system gradually decreases with age, in which multifaceted and complex changes result in hormonal imbalance and, ultimately, increased morbidity and mortality [4,98]. Aging is associated with the progression of lymphoid organ remodeling; thus, geriatric populations, possessing impaired immunity (immunosenescence), are less capable of fending off infections, autoimmune diseases, and malignancies [98,99]. During aging, dysfunctional mitochondria and oxidative damage, involving excessive production of free radicals and ROS, modulate the immune system and contribute to increased morbidity and mortality [100]. As a consequence, the impaired immune system in aging fails to prevent a variety of pathogens, including COVID-19, resulting in higher mortalities with elderly individuals aged 65 and above. Studies have shown that an imbalance between production of free radicals/ROS and protective antioxidant systems, affecting cellular oxidative damage, induces age-related complications and diseases [101]. In addition, ROS disrupts mitochondrial function and decreases the steroidogenic acute regulatory protein, a key factor that is implicated in the age-related decline of steroid hormones [4,102]. As such, oxidative damage induced by ROS is deleterious to the functional efficiency of various cellular processes, including the immune system, leading to severe COVID-19-associated complications and mortalities. 

Underlying medical conditions represent pre-existing complications and diseases, which impair the immune system and predispose aging populations, especially males, to severe COVID-19-related infections and mortalities. These medical conditions include but are not limited to obesity, diabetes, CVDs, cancers, and neurological disorders. 

### 6.1. Obesity

Obesity is a complex disease characterized by excessive accumulation of fat; it decreases the overall quality of life and increases risk for diabetes, CVDs, and certain cancers, all of which are detrimental to COVID-19 infections [42,103,104]. Obesity dampens the immune system, generates a pro-inflammatory response with increased cytokine levels, fails to prevent pathogenic threats, and results in severe COVID-19-related complications and mortalities [103]. Whereas the immune system influences host defense, adipose/fat tissue plays an important role in energy balance and homeostasis. With obesity, the immune system function in the adipose tissues become altered, as cells responsible for regulation of systemic metabolism and bodily homeostasis are exchanged for cells responsible for inflammatory responses [105]. This generates higher-than-average cytokine levels that are prone to develop COVID-19-linked difficulties. Furthermore, the overabundance of adipose tissues affects the respiratory system and limits the ability of an individual to effectively utilize oxygen to perform different cellular activities [103,106]. Cells that are not able to function at optimal levels fail to prevent COVID-19 infections, resulting in multi-organ damages with deadly outcomes.

### 6.2. Diabetes

Diabetes is a chronic condition and is broadly recognized as a plausible comorbidity that coincides with prolonged and extensive obesity. In diabetes, the pancreas produces little to no insulin, or the latter is not utilized to breakdown foods to sugar/glucose, an important process for energy metabolism and proper functioning of various physiological activities. Diabetes and obesity (leading to CVDs) are the most common comorbidities of COVID-19-associated complications [107,108,109]. It has been reported that the expression of ACE2 receptors is upregulated in diabetes, allowing for higher levels of the COVID virus binding to the host cells and resulting in progressive infections [110,111]. Diabetes also increases circulating levels of furin, which is known to assist host–COVID-19 interactions [112]. The effect of diabetes on impaired immunity is recognized as a potential mechanism for increased morbidity and susceptibility to severe COVID-19-related complications and mortalities. Diabetes inhibits the function of neutrophils in chemotaxis, phagocytosis, and intracellular microbe neutralization, leading to a hyperinflammatory state [108]. It is conceivable that hyperinflammation is connected with an increased ability of the COVID-19 virus to bind to upregulated ACE2, involving higher proliferation rates and subsequent infections of different organs, and resulting mortalities. 

### 6.3. CVDs

CVD, the leading cause of morbidity and mortality worldwide, often evolves due to accumulation of fat and cholesterol in the arterial intima, a condition known as atherosclerosis [4,96,113,114]. While numerous processes, involving dysfunctional macrophage cholesterol homeostasis, contribute to the initiation and progression of atherosclerotic lesions, elimination of excess lipids/cholesterol is crucial in limiting plaque stability and regression of atherosclerosis [96,115,116,117]. We have reported that vitamin A/retinoid signaling effectively enhances macrophage cholesterol efflux, which is considered as a fundamental process in stabilizing and/or regressing CVDs [4,96,118]. Noteworthily, obesity is a major risk factor of CVDs, which generally display severe COVID-19-related complications. It is established that the COVID-19 virus binds to ACE2, which is expressed in many tissues, including lungs and heart, thus contributing to respiratory illnesses, as well as inflammatory processes such as myocarditis and pericarditis [119,120]. In accordance with this, patients with CVDs, involving impaired immune health, are at a higher risk to develop severe COVID-19 infections. 

### 6.4. Cancers

Cancer is a multifactorial condition with aberrant growth of cells. Alterations in gene expression result in uncontrolled growth of cells involving tumor progression [121,122]. Human genome is mostly transcribed but not translated, and gene amplification, involving oncogene activation, is a key event in the growth and development of cancers. Underlying medical conditions such as cancers show worse outcomes from COVID-19-associated infections [123,124]. Immunoediting is a dynamic process in which a host’s immune system works to recognize and destroy cancerous cells before they pose a risk [125]. Malfunction in the immune system, caused by diverse factors and/or processes, leads to an inability to distinguish and neutralize pathogens posing a greater risk for developing cancers. Specific cancer therapies (chemotherapy, radiotherapy, and surgical recoveries) can also lower an individual’s immune health and can further pose risks to cancer patients that contract COVID-19 [126,127]. Because of their impaired immune systems, cancer patients often develop various comorbidities such as diabetes, obesity, and CVDs [128,129]. It has been reported that ACE2 expression is upregulated in various cancerous tissues, which allow COVID-19 virus to bind and enter host cells effectively and result in higher mortalities [130,131]. Consequently, the effect of COVID-19 has been harsh in cancer patients as they frequently possess the weakest immune system either by cancer itself or due to associated therapies and therefore develop radical complications with a lower incidence of survival. 

### 6.5. Neurological Diseases

Neurological disorders generally affect cognitive, behavioral, and social skills of a person’s ability to act independently. Several hundred neurological complications/diseases have been identified, which include Alzheimer’s disease (AD), stroke, Huntington’s disease (HD), epilepsy, and Parkinson’s disease (PD), all of which are vulnerable to COVID-19 infections and related consequences. Furthermore, inflammation in the brain can bring about seizures, delirium, coma, and other neurological manifestations [97,132,133,134]. Of note, AD is the most common cause of dementia, which results in neuronal cell death due to cerebrovascular dysfunction [135]. Accumulation of amyloid-β precursor protein and Tau in the brain are the pathological hallmarks of AD, a condition most prevalent in elderly men and women [136,137]. There is increasing evidence that patients diagnosed with AD have significantly higher levels of ACE2 receptors in the brain, in comparison to aged individuals without AD, implicating that AD patients are more prone to COVID-19 infections [138]. HD is an inherited disorder that is associated with a wide spectrum of symptoms, including cognitive decline and involuntary movements. Mutations in the huntingtin (a key protein in embryonic development and brain function) gene, located at chromosome 4, play central roles in HD. Another neurological disorder, PD, affects middle-aged to elderly people with tremors, muscular rigidity, and balance and movement problems and is influenced by genetic and environmental factors [139,140,141]. PD degenerates dopamine-producing neurons in the brain, thus lowering the levels of dopamine, a neurotransmitter important for movement and coordination [142]. Aging populations generally possess impaired immune responses, which are associated with numerous health complications along with a host of pathologies; however, males, in comparison to females, are predisposed to severe COVID-19 infections, hospitalizations, and mortalities.

## 7. Therapeutic Approaches for the Prevention and Treatment of COVID-19

The prevention of diseases is paramount to modern healthcare, and this concept carries over to COVID-19, for which no precise treatment is currently available. This hostile virus has infected hundreds of millions of people, along with countless numbers of mortalities, in which COVID-19 is destructive to elderly individuals and people with underlying medical conditions, especially men (in comparison to women) [75,143]. It should be noted, however, that COVID-19 is susceptible to individuals of all age groups who possess impaired immunity. As such, maintenance of a robust immune system is considered to be the first line of prevention not only for COVID-19 but also for other invading pathogens [41]. For limiting the spread of COVID-19, the WHO, the governments of many counties, and their disease-prevention and -control centers have advocated several measures and/or practices such as face coverings, sanitization, avoidance of parties and/or gatherings, and physical distancing, in addition to vaccination [144]. Many of these practices limit and/or decrease illnesses because COVID-19 infection spreads through aerosol droplets from infected individuals reaching the nose, eyes, and mouth after being expelled through coughing or sneezing (Table 1). Thus, the use of face masks is effective to prevent the spread of COVID-19 infections. Studies of viral infections such as influenza have reported that physical distancing is effective at limiting disease spread, a scenario certainly influential for the protection of COVID-19 infections. The most effective preventive measure for deadly contagious diseases, including COVID-19, is vaccination, which fundamentally boosts the immune system, especially in susceptible populations [145,146]. Vaccines allow for preemptive development of memory B- and T-cells to neutralize pathogens; however, the efficacy of vaccines depends on age, existing immunity, underlying medical conditions, and/or and immunological disorders. For COVID-19, many countries have established and urgently approved vaccines for eligible individuals to curb the infections [147]. In the United States, three vaccines have been developed by pharmaceutical companies and received such approval, i.e., Pfizer-BioNTech, Moderna, and Johnson & Johnson, and are highly efficacious at preventing illness with certain age grouped individuals [148]. 

Vaccines are the most effective preventive measures against COVID-19 (and other pathogens); however, several agents have been postulated based on their pharmacologic effects, which play fundamental roles in the prophylaxis and/or improvement of COVID-19-associated symptoms. These agents, many of them occurring naturally, display a variety of properties, including anti-oxidative (e.g., vitamin C, trans-resveratrol, kale, and pecans), anti-inflammatory (e.g., miodesin, berries, nuts, and curcumin), and immunomodulatory effects that can be either endogenous (e.g., hormones, cytokines, and growth factors) or exogenous (e.g., nutritional supplements such as vitamins, zinc, selenium, transfer factors, and spirulina) in preventing and/or ameliorating the severity of COVID-19-related infections and complications [149,150]. Many of these compounds, including vitamins, phytochemicals, and nutraceuticals, directly strengthen the immune system for defending invading pathogens. Additionally, the immunomodulatory effects of glucans, especially the natural glycophosphopeptical compound AM3, have been proposed as an adjuvant therapeutic agent against COVID-19 [151]. Furthermore, melatonin, a bioactive compound, with a number of health-promoting activities, along with anti-inflammatory, anti-oxidative, and immunomodulatory properties, has been reported to regress/limit severe symptoms and complications in COVID-19 patients [150]. As such, agents that strengthen the immune system could be targeted for potential therapies in the prevention of COVID-19-associated infections. 

**Figure 5:**
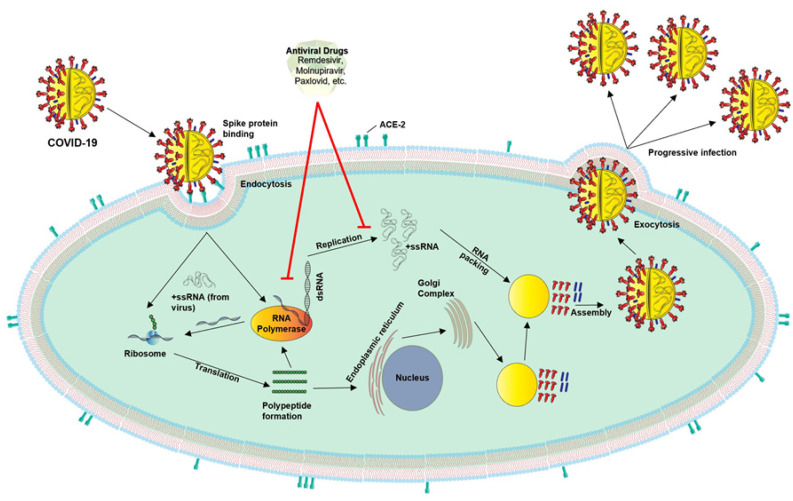
A diagrammatic representation illustrating cellular interaction of COVID-19; its replication process, assembly, release; and potential treatment options with antiviral drugs. COVID-19 virus enters the host through various means such as the inhalation of aerosol droplets. Then, the S1 subunit of the spike glycoprotein binds with the ACE-2 receptors (top left), which allows for viral ++ssRNA to be released into the host cell, where it employs cellular machinery to produce its RNA polymerase, its genetic material, and proteins needed for the viral capsule (middle-to-bottom left). Excluding the enzymatic and genomic components, other components are processed using endoplasmic reticulum and the Golgi complex (bottom middle). The viral ++ssRNA copies are then brought together and assembled with other viral components (middle bottom right). The newly produced virus exits the cell through exocytosis, where it can go on to infect more cells (top right). Antiviral drugs (top middle) such as Remdesivir, Molnupiravir, and Paxlovid are thought to disrupt this replication process, thus inhibiting disease progression.

COVID-19 infections are also targeted with certain treatment plans that have been urgently approved and implemented to diminish severity and fatal outcomes. Since COVID-19 infections are generally associated with excessive and persistent inflammation in the lungs and other tissues, leading to multi organ failures and deaths, anti-inflammatory interventions have been introduced in managing COVID-19 complications, utilizing different antagonists [13]. For replication (Figure 5), COVID-19 virus uses RNA polymerase, proteases, methyltransferase, and exoribonuclease; thus, several antiviral and antiretroviral drugs are being studied and/or have been used to treat severe COVID-19-related complications [23,24,152,153,154]. These drugs include Ribavirin (Tribavirin), Ritonavir (Lopinavir/Norvir), Remdesivir (Veklury), Nelfinavir (Viracept), Umifenovir (Arbidol), and Chloroquine and Hydroxychloroquine (antimalarial drugs), which have been urgently approved to treat COVID-19 patients [152,155]. Both Remdesivir and Chloroquine received EUA by the FDA for treatment of COVID-19, and they, especially Remdesivir, were found to reduce COVID-19-related complications in certain age grouped people [19,20]. Similarly, Umifenovir was reported to have moderate effects in the management of severe COVID-19 infections [25]. Recently, Merck and Co., in collaboration with Ridgeback Therapeutics, have developed an antiviral oral drug, named Molnupiravir, for the treatment of COVID-19 patients, which shows promising effects against infections and reduces hospitalizations and fatal outcomes by 50%. Of note, the European Medicines Agency has issued emergency authorization of Molnupiravir (also known as Lagevrio or MK4482) for adult COVID-19 patients suffering with increased complications and illnesses [156,157]. The FDA has also recently approved an antiviral oral drug, developed by Pfizer Inc., known as Paxlovid (Nirmatrelvir/Ritonavir), which reduces the risk of hospitalizations and deaths by 89% [158,159]. In addition to antiviral drugs, a number of monoclonal antibodies, generated by different pharmaceutical companies, i.e., Bamlanivimab + Estesevimab (Eli Lilly and Co.), Casirivimab + Imdevimab (Regeneron), and Sotrovimab (GlaxoSmithKline plc), respectively, have also been used for the treatment of COVID-19 patients [21,22]. Regeneron’s antibody cocktail treatment has received a sponsorship by the WHO to be used in groups that are not developing natural immunity to COVID-19 and who may be at higher risk for severe COVID-19 illnesses. Although certain options have been developed for the management of COVID-19, more time is required for the targeted therapies in the prevention and treatment of this deadly disease. Overall, the contribution of this study includes an update on various aspects concerning COVID-19, such as risk factors, disease pathogenesis, host–pathogen interacting mechanisms, and therapeutic strategies for the prevention and/or treatment of COVID-19. Additionally, the importance of immunomodulation as a preventive medicine is emphasized, for evading severe COVID-19-associated complications.

## 8. Limitations

Due to lack of the precise evidence and methodological heterogeneity, the results highlighted in this review need to be carefully interpreted. Articles used for this review connecting COVID-19 are from the late 2019 or early 2020; however, many of them had limited data because of the unprecedented nature of the pandemic. While efforts were made to search for and designate relevant articles to the theme of this study, it is possible and highly likely that all pertinent articles were not evaluated and therefore not given equal opportunity to contribute to the knowledge within this review. Another limitation lies within the short timeframe, in which articles were searched for between early 2020 and January 2022. The ever-evolving nature of the COVID-19 pandemic reveals that constant information is being discovered and published. Even so, this study provides an overview of the current literature connecting COVID-19 infections, symptomatology, molecular pathogenesis, and potential therapeutic approaches, in which caution has been taken to conscientiously discuss and/or analyze the importance of healthy immunity in the management of this hostile disease.

## 9. Conclusions and Challenges

COVID-19 is an ever-emerging multi organ system disorder, in which this virus enters host cells by binding to the ACE2 receptors. Airborne transmission is the principal mode of infection for the spread of COVID-19 [11,12]. The pathophysiological manifestations of this disease include aberrant respiratory distress, hypoxia, lung injury, inflammation, and a cytokine storm [13,14]. Patients afflicted with COVID-19 display diverse conditions, ranging from asymptomatic to severe symptoms, which involve aging and age-related comorbidities; sex and gender differences; and underlying medical conditions such as cancers, diabetes, autoimmune and inherited diseases, and neurological disorders [25,37,160]. Importantly, geriatric populations, especially men in comparison to women, are radically affected by severe COVID-19 infections, along with mortalities (Figure 4), underscoring the importance of a boosted immune health in the fundamental protection from this deadly disease. This has been affected by a lack of knowledge regarding the mechanisms of action of COVID-19, its genomic variants (especially Delta and Omicron), and effective prevention and treatment paradigms. Therefore, a healthy immune system is considered to be an essential tool in preventing people from COVID-19 contraction or other environmental factors, bacteria, and viruses. Eating a balanced diet with vitamins and minerals, along with healthy lifestyle, involving a strengthened immune system, is instrumental in diminishing COVID-19 infections, hospitalizations, and mortalities [41,143]. Hence, nutritional therapy, involving immunomodulation, is the first line of a body’s natural defense and could be considered as a priority of preventive medicine today in the management of COVID-19.

Despite the significance of the immune system, knowledge of the molecular pathogenesis of COVID-19, involving host–pathogen interacting mechanism(s), symptomatology, and transmission, is crucial towards developing a precise response towards targeting/limiting this deadly disease [161]. Advances in genomic and proteomic technologies have provided insights into the mechanisms that facilitate a better understanding of COVID-19 pathogenesis, information on which is the driving force for potential targets in controlling this disease. While vaccines prevent people from contracting COVID-19, effective drugs (e.g., antiviral, antibodies, antiretroviral, and others), along with little to no side effects, are vital for the treatment of this devastating disease [23,24]. As more discoveries, and additional patient/clinical data, become available, obtaining a precise understanding of the biological activities of COVID-19 and its variants along with their pathogenesis is the key to developing therapeutic strategies for the management of COVID-19 (Figure 5). Regardless of various approaches, including the emergence of therapeutic paradigms, healthy immunity is, undoubtedly, and will always be, a unique and highly relevant measure in the prevention of COVID-19, and other invading pathogens.

## Figures and Tables

**Table 1:** Relative levels of various risk factors for COVID-19 infections.

Risk Factors	Risk Levels		
	Low	Medium	High
Women		+	
Men			++
Ages (0–24 years)	+		
Ages (25–64 years)		+	
Ages (65+ years)			+++
Hotel stays (<2 nights)			+
Visiting museums or libraries	+	+	
Public playground		+	
Attending dinner parties		+	+
Shopping at malls			+
School/daycare		+	+
Indoor in-person jobs		+	
Haircut/salon visit		+	
Wedding			+
Restaurant eating			+
Working out at gyms			+
Attending sporting events		+	
Drinking at bars			+
Attending concerts			++
Public pool			+
Visiting friends/relatives			+
Going to movie/theater			+
Parties			++
Travels (bus/train/plain)			+
Public transport			++
Attending in-person classes		+	+
Cruise travel			++
Hospital visit		+	
Graduation parties			++

‘+’, ‘++’, and ‘+++’ represent risk at different levels (+, low; +++, high).

## Data Availability

Not applicable.
